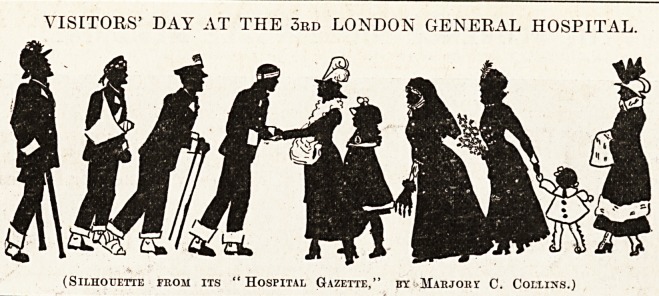# Schools of Massage: National Hospital for the Paralysed and Epileptic

**Published:** 1916-03-04

**Authors:** 


VISITORS' DAY AT THE 3rd LONDON GENERAL HOSPITAL.

(Silhouette from its "Hospital Gazette," by Marjory C. Collins.)

### SCHOOLS OF MASSAGE.

### National Hospital for the Paralysed and Epileptic.

There is no more striking feature in the development of voluntary hospitals during the last fifteen years than the evolution of the massage training school, which has become an important department of most of our great hospitals both in London and the provinces. Institutions which till recently seemed to be fully occupied with the tasks of caring for the sick and at the same time rendering invaluable service to the public by affording facilities for the training of medical students and of nurses, have now opened their wards and out-patient departments to yet another class of learner; and thus have made possible an efficient training in massage and remedial exercises, which hitherto was very difficult to obtain in this country.

During the opening years of this century massage treatment was ordered by physicians and surgeons for a certain proportion of their patients; but in a general hospital the number of patients under this treatment was not more than could be dealt with by one masseuse, who was usually non-resident and often undertook private cases in addition to her hospital work.

The National Hospital being one in which massage had for many years been considered one of the most important means of treatment, differed from general hospitals in that the nurses were trained to apply this treatment to the women patients. It was regarded as part of their training, and a certificate stating that they had gained proficiency in that art was given them at the end of a certain period—viz., in the case of previously trained nurses 1½ years, and of probationers three years' training, of which the last year was chiefly occupied in the study of massage. In the days before a central examining body existed this school was one of the very few institutions on whose training medical men felt able to place reliance, and there was a great demand for the services of nurses trained in massage at this hospital. The certificate still has considerable value with physicians, who are aware of the ample opportunities for gaining proficiency afforded to the nursing staff at this hospital; and there have always been many general-trained nurses glad to avail themselves of the chance of obtaining without expense training in a branch of nursing which would enable them to earn considerably higher salaries as private nurses.

As the certificate of the Incorporated Society of Trained Masseuses gradually became a recognised standard of efficiency it was found desirable for the nurses to enter for this examination in addition to that held at their own school; and in 1904 the step was taken, which has since been followed by nearly all the great London hospitals, of allowing non-resident lady pupils, who had received no previous training in nursing, to attend a six months' course of instruction in massage and remedial exercises at the hospital.

This arrangement still continues; at the present time there are, in addition to twenty nurses and probationers who are being taught massage, twenty lady students who share with them the instruction in anatomy, physiology, the theory and practice of massage, active and passive movements, Swedish exercises, bandaging and the use of splints, and such nursing details as are required of the masseuse or masseur. The course conforms with the requirements of the Incorporated Society of Trained Masseuses, and pupils of the school are eligible as candidates for the Incorporated Society's examinations, which are held shortly after the examination of the school.

The outbreak of the present war and the consequent admission of wounded soldiers to the wards has had the effect of widening the range of cases for treatment, and the setting free of the male nurses for active service has resulted in the massage work in the other male wards being undertaken by nurses and pupils. Needless to say, the work is carried on under skilled supervision, for, in addition to the instructress, Miss Hawkins, who attends daily, a resident massage sister is responsible for the careful and regular execution of massage treatment.

The medical cases for which massage treatment is prescribed at the National Hospital are varied, and the massage pupils studying at this school should have a thorough knowledge of their work so far as medical and chronic cases are concerned, for they constitute a very large proportion of the cases for which massage is required. The students share with the nurses the privilege of attending lectures by the honorary staff on the diseases which they are called upon to treat. The gymnastic apparatus and mechanical exercisers, and the large and airy rooms in which treatment is given, allowing ample space for dealing with large numbers of patients and massage operators, are marked features of this training school.

## Figures and Tables

**Figure f1:**